# Renal status in elderly patients with type 2 diabetes

**DOI:** 10.1007/s10157-019-01792-9

**Published:** 2019-09-23

**Authors:** Kazunaga Takamatsu

**Affiliations:** Takamatsu Medical Clinic, 2-5-48, Ookawasuji, Kochi-City, 780-0052 Kochi Japan

**Keywords:** Renal disorder, Elderly patients, Type 2 diabetes, Chronic kidney disease

## Abstract

**Background:**

In Japan, there has been a remarkable increase in the incidence of type 2 diabetes in elderly patients. This study aimed to clarify the renal status in elderly patients with type 2 diabetes.

**Participants and methods:**

There were 978 patients with type 2 diabetes who were classified into three groups: Group 1 (aged < 65 years of age), Group 2 (65–74 years of age), and Group 3 (≥ 75 years of age). Estimated glomerular filtration rate (eGFR) and urinary albumin level were measured. Moreover, the frequencies of each stage of chronic kidney disease for each group were determined, and differences among the three groups were analyzed.

**Results:**

The mean eGFR in Group 3 was 63.2 ± 19.1 mL/min/1.73 m^2^, which was lower than those in Group 1 (83.3 ± 22.8 mL/min/1.73 m^2^) and Group 2 (72.0 ± 19.4 mL/min/1.73 m^2^). The percentage of low eGFR (< 60 mL/min/1.73 m^2^) with normo- and microalbuminuria in Group 3 was 31.9%, which was higher than the percentages observed in Group 1 (7.1%) or Group 2 (16.1%). Diabetic macroangiopathy was frequently observed in these patients. The frequency of low eGFR with proteinuria was 10.2%. In this group, diabetic micro- and macroangiopathies were frequently noted.

**Conclusion:**

In elderly patients with type 2 diabetes, renal dysfunction is characterized by low eGFR with normo- and microalbuminuria. In this group, macroangiopathy was more common than microangiopathy. The elderly patients with diabetes with low eGFR with proteinuria frequently had micro- and macroangiopathies.

## Introduction

According to the National Health and Nutrition Survey, the incidence of diabetes increases with age [1]. As the number of elderly patients with diabetes increases, there are increasing needs to understand the characteristics of abnormal glucose metabolism and diabetic complications including nephropathy in these patients. With regard to renal damage in elderly patients with diabetes, the high incidence of chronic kidney disease (CKD) (reduced renal function), microalbuminuria and macroangiopathy including coronary heart disease (CHD), and rapid decline in renal function have been reported [2–7].

The Japanese Society of Nephrology has established a system for staging CKD. These stages are classified as A1 (< 30 mg/g Cr), A2 (30–299 mg/g Cr), and A3 (≥ 300 mg/g Cr) based on urinary albumin concentration (U-Alb) and G1 (≥ 90 mL/min/1.73 m^2^), G2 (60–89 mL/min/1.73 m^2^), G3a (45–59 mL/min/1.73 m^2^), G3b (30–44 mL/min/1.73 m^2^), G4 (15–29 mL/min/1.73 m^2^), and G5 (< 15 mL/min/1.73 m^2^) based on the estimated glomerular filtration rate (eGFR). The risks of death, end-stage renal failure, and cardiovascular mortality at each stage are indicated [8]. However, the previous studies did not evaluate the characteristics of patients divided into CKD stages [2–7].

In this study, to clarify the renal status in elderly patients with type 2 diabetes, participants were divided into three groups based on age, and the frequencies of each stage in each group were determined and compared with clinical profiles.

## Research design and methods

All patients fulfilled the following diabetes diagnostic criteria of the Japan Diabetes Society [9]: criterion 1, fasting plasma glucose level ≥ 126 mg/dL or casual plasma glucose level ≥ 200 mg/dL or plasma glucose level in 2 h of 75 g glucose tolerance test ≥ 200 mg/dL, and criterion 2, hemoglobin A1c level ≥ 6.5%. The diagnosis of diabetes mellitus was made by meeting two or more items in criterion 1 on different days or criteria 1 and 2 on the same day. The stage for each patient was determined based on the eGFR, U-Alb, and urine protein levels, and then the frequencies for each stage of CKD in each group were obtained. To characterize renal disorders among individuals aged ≥ 75 years, patients’ clinical profiles were examined and compared. Regarding patients’ clinical background, retinopathy was diagnosed by ophthalmologists. Hypertension was defined as systolic blood pressure ≥ 140 mmHg and/or diastolic blood pressure ≥ 90 mmHg and/or undergoing hypertensive treatment. Dyslipidemia was defined as low-density lipoprotein cholesterol level ≥ 140 mg/dL and/or triglyceride level ≥ 220 mg/dL and/or high-density lipoprotein cholesterol level < 40 mg/dL and/or undergoing treatment for dyslipidemia. The presence or absence of CHD, cerebrovascular disease (CVD) and peripheral artery disease (PAD) was confirmed by referring to each patient’s individual medical record. Patients with history of percutaneous coronary interventions or coronary artery bypass grafting were diagnosed as having CHD. CVD was diagnosed by specialists according to the significant findings of brain magnetic resonance imaging or computed tomography. PAD was diagnosed by specialists according to clinical symptoms and the significant findings of angiography or multi-detector computed tomography findings.

eGFR as per the modification of diet in Renal Disease study equation using the Japanese coefficient was calculated [10]. Serum creatinine, U-Alb, and urinary protein levels were measured using the enzymatic, immunoturbidimetric, and pyrogallol red methods, in the BML, a commercial laboratory facility. This study was a cross-sectional study.

Results are presented as means ± standard deviations, and all analyses were performed using the BellCurve for Excel (version 2.15, 2015). Between-group differences were determined using the χ^2^ test and Welch’s *t* test. A *p* value < 0.05 indicated statistical significance.

## Results

Clinical profiles of patients are shown in Table 1. There were 978 patients with type 2 diabetes (male/female ratio, 618/360; mean age, 66.7 ± 10.8 years) who were under treatment in the clinic from January 4, 2016 until December 30, 2016. This was a cross-sectional study.

**Table 1:** Clinical profiles of the patients

	Group 1	Group 2	Group 3
Cases	366	386	226
Age (years)	55.7 ± 7.8	69.2 ± 2.9^a^	80.2 ± 4.2^b^
Male/female ratio	265/101	239/147^a^	114/112^b^
Disease duration (years)	10.1 ± 8.9	11.2 ± 5.3^c^	15.4 ± 7.6^b^
BMI (kg/m^2^)	25.7 ± 4.3	24.1 ± 3.6^a^	23.6 ± 3.3^a^
eGFR (mL/min/1.73 m^2^)	83.3 ± 22.8	72.0 ± 19.4^a^	63.2 ± 19.1^b^
Urinary albumin (mg/g Cr)	74.0 ± 330.3^d^	60.0 ± 178.4^e^	114.9 ± 348.4^f,g^
Urinary protein (mg/g Cr)	2815.9 ± 3124.7^h^	2207.0 ± 2715.4^i^	3888.5 ± 4803.8^j^
CPG (mg/dL)	180.6 ± 87.1	167.3 ± 60.5^k^	170.7 ± 59.1
HbA1C (%)	7.3 ± 1.4	7.0 ± 1.0^l^	7.0 ± 1.0^m^
Hb (g/dL)	14.3 ± 1.5	13.7 ± 1.4^a^	13.0 ± 1.4^b^
RBC (×10^4^)	467.1 ± 47.7	447.4 ± 42.1^a^	424.2 ± 46.6^b^
T-Ch (mg/dL)	193.0 ± 34.1	184.3 ± 29.5^a^	180.7 ± 29.2^b^
TG (mg/dL)	185.6 ± 138.6	141.7 ± 81.9^a^	126.1 ± 81.9^a,n^
HDL-C (mg/dL)	51.9 ± 15.1	53.6 ± 14.5	53.4 ± 15.7
LDL-C (mg/dL)	104.0 ± 33.1	102.3 ± 26.2	101.8 ± 27.0
Uric acid (mg/dL)	5.3 ± 1.3	5.1 ± 1.3^p^	5.2 ± 1.3
sBP (mmHg)	128.6 ± 13.8	130.7 ± 11.3^p^	129.6 ± 9.8
dBP (mmHg)	76.0 ± 7.6	74.5 ± 5.9^q^	72.7 ± 5.3^b^
Retinopathy (yes/no)	51/315	114/272^a^	74/152^b^
Hypertension (yes/no)	199/167	241/145^a^	147/79^a^
ARB/ACE-I (yes/no)	144/222	164/222	97/129
CCB (yes/no)	107/259	148/238^s^	106/120^a,s^
Dyslipidemia (yes/no)	204/162	198/188	122/104
Statin (yes/no)	112/254	157/229^a^	113/113^a,t^
Fibrate (yes/no)	46/320	10/376^a^	6/220^a^
CHD (yes/no)	18/348	35/351^u^	47/179^b^
CVD (yes/no)	30/336	39/347	32/194^v^
PAD (yes/no)	9/357	7/379	17/209^w,x^
Therapy (D/O/I)	70/274/22	59/285/42^a^	38/165/23^a^

*BMI* body mass index, *eGFR* estimated glomerular filtration rate, *CPG* casual plasma glucose, *HbA1c* hemoglobin A1c, *Hb* hemoglobin, *RBC* red blood cell, *T-Ch* total cholesterol, *TG* triglyceride, *HDL-C* high-density lipoprotein cholesterol, *LDL-C* low-density lipoprotein cholesterol, *sBP* systolic blood pressure, *dBP* diastolic blood pressure, *ARB* angiotensin II receptor blocker, *ACE-I* angiotensin-converting-enzyme inhibitor, *CCB* calcium channel blocker, *CHD* coronary heart disease, *CVD* cerebrovascular disease, *PAD* peripheral artery disease, *Therapy (D/O/I)* therapy (diet/oral agents/insulin)

^a^*p* < 0.0001 vs. Group 1

^b^*p* < 0.0001 vs. Group 1 and Group 2

^c^*p* = 0.0382 vs. Group 1

^d^*n* = 346

^e^*n* = 353

^f^*n* = 213

^g^*p* = 0.0322 vs. Group 2

^h^*n* = 20

^i^*n* = 33

^j^*n* = 13

^k^*p* = 0.0157 vs. Group 1

^l^*p* = 0.0026 vs. Group 1

^m^*p* = 0.0090 vs. Group 1

^n^*p* = 0.0153 vs. Group 2

^o^*p* = 0.0199 vs. Group 1

^p^*p* = 0.0208 vs. Group 1

^q^*p* = 0.0035 vs. Group 1

^r^*p* = 0.0012 vs. Group 1

^s^*p* = 0.0056 vs. Group 2

^t^*p* = 0.0021 vs. Group 2

^u^*p* = 0.0212 vs. Group 1

^v^*p* = 0.00142 vs. Group 1

^w^*p* = 0.0026 vs. Group 1

^x^*p* < 0.001 vs. Group 2

Participants were divided into three groups: Group 1 (< 65 years) included 366 patients (male/female ratio, 265/101; mean age, 55.7 ± 7.8 years), Group 2 (65–74 years) included 386 patients (male/female ratio, 239/147; mean age, 69.2 ± 2.9 years), and Group 3 (≥ 75 years) included 226 patients (male/female ratio, 114/112; mean age, 80.2 ± 4.2 years).

### eGFR (Table 1)

Data distributions were examined using the χ^2^ test and histograms. For each group, eGFR was normally distributed (Group 1, *p* = 0.2092; Group 2, *p* = 0.3335; Group 3, *p* = 0.5091).

The mean eGFR in Group 3 was 63.2 ± 19.1 mL/min/1.73 m^2^, which was significantly lower than those observed in Group 1 (83.3 ± 22.8 mL/min/1.73 m^2^) and Group 2 (72.0 ± 19.4 mL/min/1.73 m^2^) (*p* < 0.0001 vs. Group 1 and Group 2). The mean eGFR in Group 2 was significantly lower than that in Group 1 (*p* < 0.0001).

In men, the mean eGFR in Group 3 was 64.4 ± 18.2 mL/min/1.73 m^2^, which was significantly lower than those observed for Group 1 (83.4 ± 22.2 mL/min/1.73 m^2^) and Group 2 (73.7 ± 20.2 mL/min/1.73 m^2^) (*p* < 0.0001 vs. Group 1 and Group 2).

In women, the mean eGFR in Group 3 was 60.5 ± 18.2 mL/min/1.73 m^2^, which was significantly lower than those observed in Group 1 (89.5 ± 22.9 mL/min/1.73 m^2^) and Group 2 (73.8 ± 19.7 mL/min/1.73 m^2^) (*p* < 0.0001 vs. Group 1 and Group 2).

### G classification (Table 2, Fig. 1)

**Table 2:** CKD severity classification by group

	A1	A2	A3	Total
Group 1				
G1	89 (53/36)	33 (19/14)	4 (4/0)	126 (76/50)
G2	141 (108/33)	42 (32/10)	13 (12/1)	196 (152/44)
G3a	14 (12/2)	6 (4/2)	5 (4/1)	25 (20/5)
G3b	1 (1/0)	4 (4/0)	9 (9/0)	14 (14/0)
G4	1 (1/0)	0 (0/0)	4 (2/2)	5 (3/2)
Total	246 (175/71)	85 (59/26)	35 (31/4)	366 (265/101)
Group 2				
G1	48 (26/22)	14 (9/5)	3 (1/2)	65 (36/29)
G2	166 (103/63)	48 (31/17)	12 (9/3)	226 (143/83)
G3a	32 (16/16)	14 (9/5)	17 (14/3)	63 (39/24)
G3b	5 (2/3)	11 (7/4)	9 (5/4)	25 (14/11)
G4	0 (0/0)	0 (0/0)	7 (6/1)	7 (6/1)
Total	251 (147/104)	87 (56/31)	48 (35/13)	386 (238/148)
Group 3				
G1	9 (8/1)	5 (2/3)	1 (0/1)	15 (10/5)
G2	69 (33/36)	40 (23/17)	7 (4/3)	116 (60/56)
G3a	26 (10/16)	22 (15/7)	9 (5/4)	57 (30/27)
G3b	11 (4/7)	10 (4/6)	8 (4/4)	29 (12/17)
G4	2 (0/2)	1 (0/1)	6 (2/4)	9 (2/7)
Total	117 (55/62)	78(44/34)	31(15/16)	226 (114/112)

**Fig. 1:**
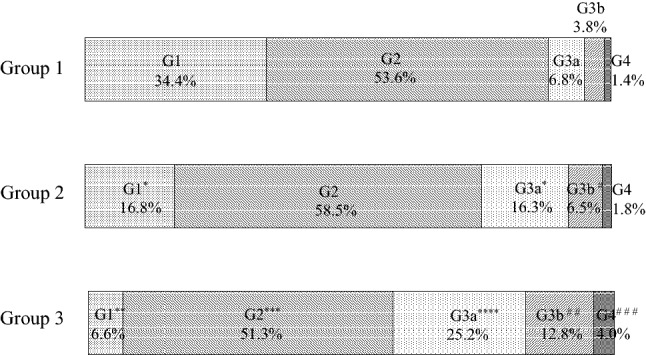
G classification frequencies by group. *p < 0.0001 vs. Group 1, **p < 0.0001 vs. Group 1 and Group2, ***p = 0.0045, ****p < 0.0001 vs. Group 1 and *p* = 0.0001 vs. Group 2, ^#^*p* = 0.0393, ^# #^*p* < 0.0001 vs. Group 1 and *p* = 0.0002 vs. Group 2, ^# # #^*p* = 0.0105 vs. Group 1 and *p* = 0.0293 vs. Group 2

The number of cases at each stage of CKD severity classification in each group is shown in Table 2. The frequencies of G classification in each group are shown in Fig. 1. The frequency of G1 in Group 3 was 6.6%, which was significantly lower than the Group 1 frequency of 34.4% and the Group 2 frequency of 16.8% (*p* < 0.0001 vs. Group 1 and Group 2). The Group 2 frequency was significantly lower than that of Group 1 (*p* < 0.0001).

The frequency of G2 in Group 3 was 51.3%, which was significantly lower than the Group 2 frequency of 58.5% (*p* = 0.0045). There was no statistically significant difference in the frequency of G2 between Group 3 and Group 1.

The frequency of G3a in Group 3 was 25.2%, which was significantly higher than those in Group 1 (6.8%) and Group 2 (16.3%) (*p* < 0.0001 vs. Group 1 and *p* = 0.0001 vs. Group 2). The frequency of G3a in Group 2 was significantly higher than that observed in Group 1 (*p* < 0.0001).

The frequency of G3b in Group 3 was 12.8%, which was significantly higher than those in Group 1 (3.8%) and Group 2 (6.5%) (*p* < 0.0001 vs. Group 1 and *p* = 0.0002 vs. Group 2). The frequency of G3b in Group 2 was significantly higher than that in Group 1 (*p* = 0.0393).

The frequency of G4 in Group 3 was 4.0%, which was significantly higher than that observed in Group 1 (1.4%) and Group 2 (1.8%) (*p* = 0.0105 vs. Group 1 and *p* = 0.0293 vs. Group 2).

The frequency of G3a + G3b + G4, corresponding to CKD, in Group 3 was 42.0%, which was significantly higher than the frequencies observed in Group 1 (12.0%) and Group 2 (24.6%) (*p* < 0.0001 vs. Group 1 and Group 2).

In men, the frequency of G3a + G3b + G4 in Group 3 was 38.6%, which was significantly higher than those in Group 1 (14.0%) and Group 2 (24.8%) (*p* < 0.0001 vs. Group 1 and Group 2). In women, the frequency of G3a + G3b + G4 in Group 3 was 45.5%, which was significantly higher than those in Group 1 (6.9%) and Group 2 (24.3%) (*p* < 0.0001 vs. Group 1 and Group 2).

### A classification (Table 2, Fig. 2)

**Fig. 2:**
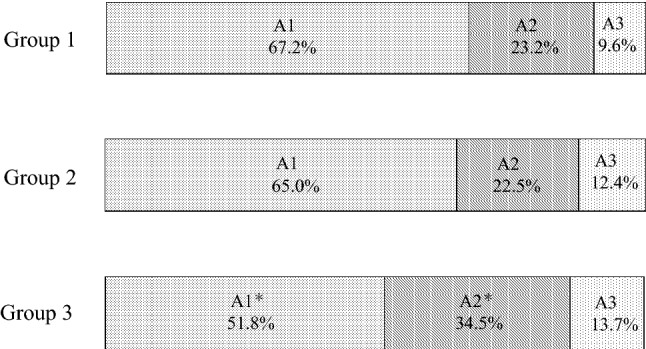
A classification frequencies by group. *p < 0.0001 vs. Group 1 and vs. Group 2

The frequencies of A classification in each group are shown in Fig. 2. The frequency of A1 in Group 3 was 51.8%, which was significantly lower than the frequency observed for Group 1 (67.2%) or Group 2 (65.0%) (*p* < 0.0001 vs. Group 1 and Group 2).

The frequency of A2 in Group 3 was 34.5%, which was significantly higher than those observed in Group 1 (23.2%) and Group 2 (22.5%) (*p* < 0.0001 vs. Group 1 and Group 2).

The frequency of A3 in Group 3 was 13.7%, which was significantly higher than that observed in Group 1 (9.6%) (*p* = 0.0209).

The frequency of A1 and G3a + G3b + G4, corresponding to CKD, in Group 3 was 17.2%, which was significantly higher than those observed in Group 1 (4.4%) and Group 2 (9.6%) (*p* < 0.0001 vs. Group 1 and *p* = 0.0001 vs. Group 2).

The frequency of A2 and G3a + G3b + G4 in Group 3 was 14.6%, which was significantly higher than those observed in Group 1 (2.7%) and Group 2 (6.5%) (*p* < 0.0001 vs. Group 1 and Group 2).

The frequency of A1 + A2 and G3a + G3b + G4 in Group 3 was 31.9% (72 cases), which was significantly higher than those observed in Group 1 (7.1%) and Group 2 (16.1%) (*p* < 0.0001 vs. Group 1 and Group 2).

The frequency of A3 and G3a + G3b + G4 in Group 3 was 10.2% (23 cases), which was significantly higher than those in Group 1 (4.9%) (*p* < 0.0001 vs. Group 1) and was not significantly different with that in Group 2(8.5%).

### Clinical profiles of G3a + G3b + G4 in Group 3 (Tables 3, 4, and 5)

**Table 3:** Clinical profiles of patients with A1 + A2 and G3a + G3b + G4 in each group

	Group 1	Group 2	Group 3
Cases	26	62	72
Age (years)	59.7 ± 5.5	70.1 ± 2.8^a^	80.6 ± 4.4^b^
Male/female ratio	22/4	34/28^c^	34/38^a^
Disease duration (years)	10.4 ± 4.8	12.7 ± 6.0	15.2 ± 7.1^a,d^
BMI (kg/m^2^)	25.8 ± 2.9	24.6 ± 3.2	23.5 ± 2.8^a,e^
eGFR (mL/min/1.73 m^2^)	49.9 ± 8.6	49.4 ± 7.6	47.1 ± 9.0
Urinary albumin (mg/g Cr)	34.8 ± 39.3	50.5 ± 66.1	52.7 ± 69.4
CPG (mg/dL)	164.7 ± 63.0	161.3 ± 51.0	172.5 ± 55.3
HbA1C (%)	7.1 ± 1.2	6.8 ± 0.7	6.9 ± 0.8
Hb (g/dL)	13.8 ± 1.3	13.5 ± 1.4	12.6 ± 1.3^b^
RBC (×10^4^)	428.0 ± 73.3	441.4 ± 45.1	413.2 ± 43.1^f^
T-Ch (mg/dL)	188.3 ± 28.7	179.8 ± 25.4	174.9 ± 28.5
TG (mg/dL)	180.6 ± 75.6	153.1 ± 90.2	134.7 ± 61.9^g^
HDL-C (mg/dL)	48.2 ± 14.9	50.1 ± 14.7	50.6 ± 15.8
LDL-C (mg/dL)	104.0 ± 23.5	99.2 ± 23.9	97.4 ± 25.1
Uric acid (mg/dL)	6.2 ± 1.4	6.0 ± 1.3	5.9 ± 1.2
sBP (mmHg)	124.8 ± 14.3	129.4 ± 11.5	128.7 ± 9.4
dBP (mmHg)	73.8 ± 7.8	73.5 ± 6.0	72.1 ± 5.7
Retinopathy (yes/no)	3/23	21/41^h^	14/58^i^
Hypertension (yes/no)	15/11	41/21	52/20
ARB/ACE-I (yes/no)	11/15	31/31	31/41
CCB (yes/no)	7/19	23/39	40/32^j,k^
Dyslipidemia (yes/no)	16/10	35/27	34/38
Statin (yes/no)	9/17	29/33	34/38
Fibrate (yes/no)	6/20	2/60^a^	4/68^a^
CHD (yes/no)	3/23	6/56	23/49^l,f^
CVD (yes/no)	2/24	9/53^a^	16/56^a^
PAD (yes/no)	1/25	2/60	8/64^m^
Therapy (D/O/I)	5/18/3	9/42/11	11/55/6^n^

*BMI* body mass index, *eGFR* estimated glomerular filtration rate, *CPG* casual plasma glucose, *HbA1c* hemoglobin A1c, *Hb* hemoglobin, *RBC* red blood cell, *T-Ch* total cholesterol, *TG* triglyceride, *HDL-C* high-density lipoprotein cholesterol, *LDL-C* low-density lipoprotein cholesterol, *sBP* systolic blood pressure, *dBP* diastolic blood pressure, *ARB* angiotensin II receptor blocker, *ACE-I* angiotensin-converting-enzyme inhibitor, *CCB* calcium channel blocker, *CHD* coronary heart disease, *CVD* cerebrovascular disease, *PAD* peripheral artery disease, *Therapy (D/O/I)* therapy (diet/oral agents/insulin)

^a^*p* < 0.0001 vs. Group 1

^b^*p* < 0.0001 vs. Group 1 and Group 2

^c^*p* = 0.0023 vs. Group 1

^d^*p* = 0.0330 vs. Group 2

^e^*p* = 0.0466 vs. Group 2

^f^*p* < 0.0001 vs. Group 2

^g^*p* = 0.0101 vs. Group 1

^h^*p* = 0.0161 vs. Group 1

^i^*p* = 0.0041 vs. Group 2

^j^*p* = 0.0033 vs. Group 1

^k^*p* = 0.0034 vs. Group 2

^l^*p* = 0.0256 vs. Group 1

^m^*p* = 0.0482 vs. Group 2

^n^*p* = 0.0271 vs. Group 2

**Table 4:** Clinical profiles of patients with A3 and G3a + G3b + G4 in each group

	Group 1	Group 2	Group 3
Cases	18	33	23
Age (years)	55.0 ± 7.4	69.6 ± 2.7^a^	81.8 ± 4.8^b^
Male/female ratio	16/2	25/8	11/12^a,c^
Disease duration (years)	14.9 ± 5.9	12.8 ± 5.7	22.0 ± 8.4^d,e^
BMI (kg/m^2^)	27.6 ± 4.6	25.7 ± 4.6	23.5 ± 3.5^f,g^
eGFR (mL/min/1.73 m^2^)	37.3 ± 11.9	42.1 ± 13.8	37.7 ± 12.8
Urinary albumin (mg/g Cr)	1776.0 ± 1793.6^h^	465.5 ± 162.0^i^	705.9 ± 480.2^j^
Urine protein (mg/g Cr) CPG (mg/dL)	3494.8 ± 3513.0^k^ 194.7 ± 97.9	2479.2 ± 2964.7^l^ 175.3 ± 55.6	3724.1 ± 5031.0^m^ 174.7 ± 52.7
HbA1C (%)	7.6 ± 1.5	7.3 ± 0.8	7.2 ± 1.0
Hb (g/dL)	13.3 ± 2.1	12.8 ± 1.5	12.3 ± 1.7
RBC (×10^4^)	433.1 ± 72.5	417.8 ± 48.4	398.5 ± 59.1
T-Ch (mg/dL)	175.6 ± 29.0	173.5 ± 28.5	175.8 ± 27.7
TG (mg/dL)	207.6 ± 194.7	145.4 ± 60.6	151.6 ± 84.8
HDL-C (mg/dL)	49.1 ± 14.8	52.2 ± 18.9	46.5 ± 16.6
LDL-C (mg/dL)	104.3 ± 44.5	105.1 ± 26.4	99.0 ± 22.6
Uric acid (mg/dL)	6.3 ± 1.3	5.8 ± 1.4	6.0 ± 1.1
sBP (mmHg)	143.0 ± 17.7	134.8 ± 10.9	132.2 ± 12.5^o^
dBP (mmHg)	83.1 ± 6.9	74.5 ± 5.5^a^	72.4 ± 6.0^a^
Retinopathy (yes/no)	18/0	28/5	20/3
Hypertension (yes/no)	17/1	33/0	20/3^p^
ARB/ACE-I (yes/no)	14/4	29/4	15/8^q^
CCB (yes/no)	13/5	24/9	16/7
Dyslipidemia (yes/no)	9/9	20/13	15/8
Statin (yes/no)	6/12	17/16	15/8^r^
Fibrate (yes/no)	1/17	2/31	0/23
CHD (yes/no)	3/15	10/23	9/14
CVD (yes/no)	3/15	6/27	6/17
PAD (yes/no)	4/14	4/29	2/21^s^
Therapy (D/O/I)	2/8/8	1/18/14	3/11/9

*BMI* body mass index, *eGFR* estimated glomerular filtration rate, *CPG* casual plasma glucose, *HbA1c* hemoglobin A1c, *Hb* hemoglobin, *RBC* red blood cell, *T-Ch* total cholesterol, *TG* triglyceride, *HDL-C* high-density lipoprotein cholesterol, *LDL-C* low-density lipoprotein cholesterol, *sBP* systolic blood pressure, *dBP* diastolic blood pressure, *ARB* angiotensin II receptor blocker, *ACE-I* angiotensin-converting-enzyme inhibitor, *CCB* calcium channel blocker, *CHD* coronary heart disease, *CVD* cerebrovascular disease, *PAD* peripheral artery disease, *Therapy (D/O/I)* therapy (diet/oral agents/insulin)

^a^*p* < 0.0001 vs. Group 1

^b^*p* < 0.0001 vs. Group 1 and Group 2

^c^*p* = 0.0013 vs. Group 2

^d^*p* = 0.0035 vs. Group 1

^e^*p* < 0.0001 vs. Group 2

^f^*p* = 0.0036 vs. Group 1

^g^*p* = 0.0498 vs. Group 2

^h^*n* = 5

^i^*n* = 7

^j^*n* = 13

^k^*n* = 13

^l^*n* = 26

^m^*n* = 10

^n^*p* = 0.00426 vs. Group 1

^o^*p* = 0.0373 vs. Group 1

^p^*p* = 0.0261 vs. Group 2

^q^*p* = 0.0063 vs. Group 2

^r^*p* = 0.0045 vs. Group 1

^s^*p* = 0.0417 vs. Group 1

In comparing the clinical profiles of A1 (34 cases) and A2 (39 cases) in G3a + G3b + G4 of Group 3, women exhibited relative high frequencies (male/female ratio, 19/14 vs. 14/25, *p* = 0.0023), and PAD was less frequent (2/31 vs. 6/33, *p* = 0.0147) in A2. However, there were no differences between two groups in the mean age, disease duration, HbA1c level, and the frequencies of retinopathy, CHD, CVD and macroangiopathy (80.4 ± 3.8 vs. 80.7 ± 4.9 years old, 14.7 ± 6.2 vs. 15.5 ± 8.1 years, 7.0 ± 0.8 vs. 6.8 ± 0.8%, 17.9 vs. 21.2%, 28.2 vs. 36.3%, 25.6% vs. 18.2%, 69.2 vs. 60.6%). Therefore, A1 and A2 clinical profiles were grouped for comparison with other groups.

In patients with A1 + A2 and G3a + G3b + G4, clinical profiles of each group were compared (Table 3). There were significantly more women in Group 3 than Group 1 (male/female ratio, 34/38 vs. 22/4, *p* < 0.0001). The average disease duration in Group 3 was significantly longer than those observed in Groups 1 and 2 (15.2 ± 7.1 years vs. 10.4 ± 4.8 years, *p* < 0.0001, vs. 12.7 ± 6.0 years, *p* = 0.0330). The frequency of retinopathy in Group 3 was significantly lower than that in Group 2 (19.4% vs. 33.9%, *p* = 0.0041). The frequency of CHD in Group 3 was significantly higher than those in Groups 1 and 2 (31.9% vs. 11.5%, *p* = 0.0256, vs. 9.7%, *p* < 0.0001). The frequencies of CVD and PAD in Group 3 were significantly higher than those in Group 1 (22.2% vs. 7.7%, *p* < 0.001, 11.1% vs. 3.8%, *p* = 0.0482). The frequency of CHD, CVD, and PAD as manifestations of macroangiopathy was significantly higher in Group 3 compared to those in Groups 1 and 2 (65.3% vs. 23.1%, *p* < 0.0001, vs. 27.4%, *p* < 0.0001).

In patients with A3 and G3a + G3b + G4, the clinical profiles of each group were compared (Table 4). There were significantly more women in Group 3 than in Groups 1 and 2 (male/female ratio, 11/12 vs. 16/2, *p* < 0.0001, vs. 25/8, *p* = 0.0013). The disease duration in Group 3 was significantly longer than those observed in Groups 1 and 2 (22.0 ± 8.4 vs. 14.9 ± 5.9 years, *p* = 0.0035, vs. 12.8 ± 5.7 years, *p* = 0.0013). There was no difference in the frequency of retinopathy among the three groups. The frequency of hypertension in Group 3 was significantly lower than that in Group 2 (87.0% vs. 100%, *p* = 0.0261). There were no differences in the frequencies of dyslipidemia, CHD, and CVD among the three groups. The frequency of PAD in Group 3 was significantly lower than that in Group 1 (8.7% vs. 22.2%, *p* = 0.0417). There was no difference in the frequency of macroangiopathy in the three groups.

In patients with G3a + G3b + G4, the clinical profiles were compared between those with A1 + A2 and A3 (Table 5). Systolic blood pressure was lower in A1 + A2 than in A3 in Group 1 (124.8 ± 14.3 vs. 143.0 ± 17.7 mmHg, *p* < 0.0001) and Group 2 (129.4 ± 11.5 vs. 134.8 ± 10.9 mmHg, *p* = 0.0130). There was no difference in systolic blood pressure in A1 + A2 and A3 in Group 3. In the frequency of retinopathy and hypertension, A1 + A2 was less frequent than A3 in all groups (11.5% vs. 100%, 33.9% vs. 84.8%, 19.4% vs. 87.0%, all *p* < 0.0001, 57.6% vs. 94.4%, 66.1% vs. 100%, 72.2% vs. 87.0%, all *p* < 0.0001). The frequency of CHD was lower in A1 + A2 than in A3 in Group 2 (9.7% vs. 30.3%, *p* < 0.0001), and the frequency of PAD was lower in A1 + A2 than in A3 in Groups 1 and 2 (3.8% vs. 22.2%, *p* = 0.0242, 3.2% vs. 12.1%, *p* = 0.0319). The frequency of macroangiopathy was lower in A1 + A2 than in A3 in Groups 1 and 2 (23.1% vs. 55.6%, 27.4% vs. 60.6%, both *p* < 0.0001), but no difference was observed in Group 3. In the frequency of insulin treatment, A1 + A2 was less frequent than A3 in all groups (11.5% vs. 44.4%, *p* = 0.0155, 17.7% vs. 42.4%, *p* < 0.0001, 8.3% vs. 39.1%, *p* < 0.0001).

**Table 5:** Clinical profiles of patients with A1 + A2 and A3 in G3a + G3b + G4 in each group

	Group 1		Group 2		Group 3	
	A1 + A2	A3	A1 + A2	A3	A1 + A2	A3
Cases	26	18	62	33	72	23
Age (years)	59.7 ± 5.5	55.0 ± 7.4	70.1 ± 2.8	69.6 ± 2.7	80.6 ± 4.4	81.8 ± 4.8
Males/females	22/4	16/2	34/28^g^	25/8	34/38	11/12
Disease duration (years)	10.4 ± 4.8^a^	14.9 ± 5.9	12.7 ± 6.0	12.8 ± 5.7	15.2 ± 7.1^m^	22.0 ± 8.4
BMI (kg/m^2^)	25.8 ± 2.9	27.6 ± 4.6	24.6 ± 3.2	25.7 ± 4.6	23.5 ± 2.8	23.5 ± 3.5
eGFR (ml/minute/1.73 m^2^)	49.9 ± 8.6^b^	37.3 ± 11.9	49.4 ± 7.6	42.1 ± 13.8	47.1 ± 9.0^n^	37.7 ± 12.8
Urine albumin (mg/g Cr)	34.8 ± 39.3	1776.0 ± 1793.6^c^	50.5 ± 66.1	465.5 ± 162.0^h^	52.7 ± 69.4	705.9 ± 480.2^o^
Urine protein (mg/g Cr)	–	3494.8 ± 3513.0^d^	–	2479.2 ± 2964.7^i^	–	3724.1 ± 5031.0^p^
CPG (mg/dl)	164.7 ± 63.0	194.7 ± 97.9	161.3 ± 51.0	175.3 ± 55.6	172.5 ± 55.3	174.7 ± 52.7
HbA1C (%)	7.1 ± 1.2	7.6 ± 1.5	6.8 ± 0.7^j^	7.3 ± 0.8	6.9 ± 0.8	7.2 ± 1.0
Hb (g/dl)	13.8 ± 1.3	13.3 ± 2.1	13.5 ± 1.4	12.8 ± 1.5	12.6 ± 1.3	12.3 ± 1.7
RBC (×104)	428.0 ± 73.3	433.1 ± 72.5	441.4 ± 45.1	417.8 ± 48.4	413.2 ± 43.1	398.5 ± 59.1
T-Ch (mg/dl)	188.3 ± 28.7	175.6 ± 29.0	179.8 ± 25.4	173.5 ± 28.5	174.9 ± 28.5	175.8 ± 27.7
TG (mg/dl)	180.6 ± 75.6	207.6 ± 194.7	153.1 ± 90.2	145.4 ± 60.6	134.7 ± 61.9	151.6 ± 84.8
HDL-C (mg/dl)	48.2 ± 14.9	49.1 ± 14.8	50.1 ± 14.7	52.2 ± 18.9	50.6 ± 15.8	46.5 ± 16.6
LDL-C (mg/dl)	104.0 ± 23.5	104.3 ± 44.5	99.2 ± 23.9	105.1 ± 26.4	97.4 ± 25.1	99.0 ± 22.6
Uric acid (mg/dl)	6.2 ± 1.4	6.3 ± 1.3	6.0 ± 1.3	5.8 ± 1.4	5.9 ± 1.2	6.0 ± 1.1
sBP (mmHg)	124.8 ± 14.3^b^	143.0 ± 17.7	129.4 ± 11.5^k^	134.8 ± 10.9	128.7 ± 9.4	132.2 ± 12.5
dBP (mmHg)	73.8 ± 7.8^b^	83.1 ± 6.9	73.5 ± 6.0	74.5 ± 5.5	72.1 ± 5.7	72.4 ± 6.0
Retinopathy (yes/no)	3/23^b^	18/0	21/41^g^	28/5	14/58^q^	20/3
Hypertension (yes/no)	15/11^b^	17/1	41/21^g^	33/0	52/20^q^	20/3
ARB/ACE-I (yes/no)	11/15^b^	14/4	31/31^g^	24/9	31/41^q^	15/8
CCB (yes/no)	7/19^b^	13/5	23/39^g^	24/9	40/32^r^	16/7
Dyslipidemia (yes/no)	16/10	9/9	35/27	20/13	34/38^s^	15/8
Statin (yes/no)	9/17	6/12	29/33	17/16	34/38s	15/8
Fibrate (yes/no)	6/20^b^	1/17	2/60	2/31	4/68	0/23
CHD (yes/no)	3/23	3/15	6/56^g^	10/23	23/49	9/14
CVD (yes/no)	2/24	3/15	9/53	6/27	16/56	6/17
PAD (yes/no)	1/25^e^	4/14	2/60^l^	4/29	8/64	2/21
Therapy (D/O/I)	5/18/3^f^	2/8/8	9/42/11^g^	1/18/14	11/55/6^q^	3/11/9

*BMI* body mass index, *eGFR* estimated glomerular filtration rate, *CPG* casual plasma glucose, *HbA1c* hemoglobin A1c, *Hb* hemoglobin, *RBC* red blood cell, *T-Ch* total cholesterol, *TG* triglyceride, *HDL-C* high-density lipoprotein cholesterol, *LDL-C* low-density lipoprotein cholesterol, *sBP* systolic blood pressure, *dBP* diastolic blood pressure, *ARB* angiotensin II receptor blocker, *ACE-I* angiotensin-converting-enzyme inhibitor, *CCB* calcium channel blocker, *CHD* coronary heart disease, *CVD* cerebrovascular disease, *PAD* peripheral artery disease, *Therapy (D/O/I)* therapy (diet/oral agents/insulin)

Group 1. A1 + A2 vs. A3

^a^*p* = 0.0027

^b^*p* < 0.0001

^c^*n* = 5

^d^*n* = 13

^e^*p* = 0.0242

^f^*p* = 0.0155

Group 2. A1 + A2 vs. A3

^g^*p* < 0.0001

^h^*n* = 7

^i^*n* = 26

^j^*p* = 0.0018

^k^*p* = 0.0130

^l^*p* = 0.0319

Group 3. A1 + A2 vs. A3

^m^*p* = 0.0023

^n^*p* = 0.0033

^o^*n* = 10

^p^*n* = 13

^q^*p* < 0.0001

^r^*p* = 0.0098

^s^*p* = 0.0013

## Discussion

The data obtained represent “real-world” findings from elderly patients aged ≥ 75 years with type 2 diabetes from one clinic in a local city in Japan.

Conventionally, in elderly patients with diabetes, it has been reported that the frequencies of decreased renal function and microalbuminuria are high [2, 4–7], but there are few studies on patients aged ≥ 75 years [6, 7]. In this study, the mean eGFR in patients aged ≥ 75 years decreased sequentially from Group 1 to Group 3. Correspondingly, Group 3 exhibited more frequent G3a, G3b, and G4 classifications than the other two groups. The frequency of eGFR < 60 mL/min/1.73 m^2^ (G3a + G3b + G4) in Group 3 was 42.0%, which was higher than that observed in Groups 1 or 2. Russo et al. reported that the frequency of eGFR < 60 mL/min/1.73 m^2^ in elderly patients with diabetes aged ≥ 75 years in Italy was 44.3% [7]. The results of this study were consistent with the findings of the above-mentioned report.

In contrast, Imai et al. examined the prevalence of CKD in the general Japanese population and reported that, in both men and women, eGFR decreased with age and, in men, the prevalence was 27.7% in those aged 70–79 years and 44.6% in those aged ≥ 80 years. In women, the prevalence was 31.1% in those aged 70–79 years and 46.1% in those aged ≥ 80 years [11]. In this study, the frequencies of CKD in those aged ≥ 75 years were 38.6% in men and 45.5% in women. Although the exact age category differed, the results of this study were roughly consistent with those reported by Imai et al [11]. Further studies on decreased eGFR in elderly patients with diabetes are required to confirm the contribution of aging and diabetes.

In the study on A classification, the frequency of A2 in Group 3 was 34.5%, which was higher than in the other two groups. Regarding A2, it was reported that 33.3% of patients aged ≥ 80 years [6] and 33.7% of patients aged ≥ 75 years [7] had microalbuminuria, and the effects of aging and diabetes on microalbuminuria should be considered. The results of this study were consistent with the above-mentioned reports it was considered that aging and diabetes influenced microalbuminuria in elderly patients with type 2 diabetes.

Subsequently, using the staging of CKD, the frequency of A classification in G3a + G3b + G4, corresponding to CKD, was examined. Overall, the frequency of A1 in Group 3 was lower compared to those in the other two groups but was higher in G3a + G3b + G4. Therefore, in G3a + G3b + G4, the frequencies of A1 and A2 in Group 3 were higher than those observed in the other two groups. Based on the above-mentioned findings, it was characteristic in elderly patients with type 2 diabetes that low eGFR (< 60 mL/min/1.73 m^2^) with normo- and microalbuminuria was frequently observed. This group accounted for 31.8% of patients with type 2 diabetes aged ≥ 75 years.

It has been reported that, in elderly patients with diabetes, the frequency of macroangiopathy and hypertension and the risk of cardiovascular disease are high [2, 7], but there has been no report on the examination of patients classified by renal function and proteinuria. In the clinical profiles of patients with low eGFR with normo- and microalbuminuria, there were more women, and the disease duration was longer. Moreover, 72.2% had hypertension, 47.2% had dyslipidemia, and 65.3% had macroangiopathy. Compared with patients with low eGFR with normo- and microalbuminuria in the other two groups, the frequency of retinopathy was lower than that of the 65–74-year age group, but the frequency of macroangiopathy was higher. In this group, macroangiopathy was more common than microangiopathy. The renal lesions in this group from the above clinical profiles are complex, which are likely related to aging kidneys, nephropathy with diabetes, nephrosclerosis with hypertension, and ischemic nephropathy with aortic arteriosclerosis due to dyslipidemia.

The frequency of patients with low eGFR with proteinuria in type 2 diabetes aged ≥ 75 years was 10.2%, and there was no difference compared to that in the 65–74-year age group. In clinical profiles of this group, there were more women, and the disease duration was longer. Moreover, 87.0% had retinopathy, 87.0% had hypertension, 65.2% had dyslipidemia, and 73.9% had macroangiopathy. In patients with low eGFR with proteinuria, there was no difference in frequency of retinopathy and macroangiopathy among the three groups, and age had no influence. It is considered that diabetic nephropathy caused the main lesion, and aging kidneys, hypertensive nephrosclerosis, and ischemic nephropathy are associated with it.

In all groups with low eGFR, the frequency of retinopathy was lower in patients with normo- and microalbuminuria compared to those in patients with proteinuria. In Groups 1 and 2, the frequency of macroangiopathy was lower in patients with normo- and microalbuminuria compared to those in patients with proteinuria. In Group 3, there was no difference in the frequency of macroangiopathy between patients with normo- and microalbuminuria and those with proteinuria. In patients aged ≥ 75 years with diabetes, the principle that the progression of microangiopathy and macroangiopathy is parallel, which was found in the other two groups, is not applicable.

This study had several limitations. This was a cross-sectional study in one center, and measurement of eGFR and U-Alb was performed only once. Moreover, the duration of diabetes was longer in patients in Group 3.

## Conclusion

Using the CKD staging system, I reported the renal status in elderly type 2 diabetic patients and the frequency of macroangiopathy was associated with renal status. This report will be useful for the treatment of elderly patients with type 2 diabetes in the future.
